# Estimated work of breathing in PAV-plus ventilation in ICU patients

**DOI:** 10.1186/cc10185

**Published:** 2011-06-22

**Authors:** LP Couto, A Thompson, F Gago, R Serafim, F Saddy, CSV Barbas

**Affiliations:** 1 Hospital das Clinicas da FMUSP, São Paulo - SP, Brazil; 2 Hospital Copa D'Or Rio de Janeiro, Rio de Janeiro - RJ, Brazil

## Background

The purpose of the new PAV-plus ventilation is to guarantee a better patient ventilator synchrony allowing the measurement of respiratory system mechanics and the estimation of the patient work of breathing.

## Objective

To verify whether ICU patients recovering from acute respiratory failure can be maintained well in PAV-plus ventilation and if the PAV-plus ventilatory mode can estimate respiratory mechanics and work of breathing in ICU clinical practice.

## Methods

We studied 20 stable ICU patients that were recovering from acute respiratory failure and could be ventilated comfortably in pressure support of 15 cmH_2_O. After 20 minutes in PSV of 15 cmH_2_O we measured the tidal volume, respiratory rate, minute ventilation, PaCO_2 _and asked the patients to give a note from 0 to 10 on a visual comfort scale. Then, we changed the patients to PAV-plus ventilation with 65% support and after 20 minutes we measured the same mentioned parameters plus the respiratory system compliance, resistance and the patients work of breathing. The same procedure was made after changing the patients to PAV-plus ventilation of 50% support. We established the association between the estimated work of breathing by the ventilator and the measured respiratory parameters (*P *< 0.05).

## Results

**Figure 1:**
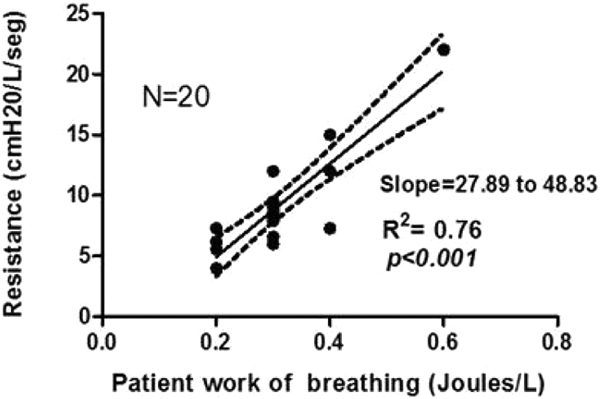
**Correlation between resistance and WOB in PAV-plus**.

**Figure 2:**
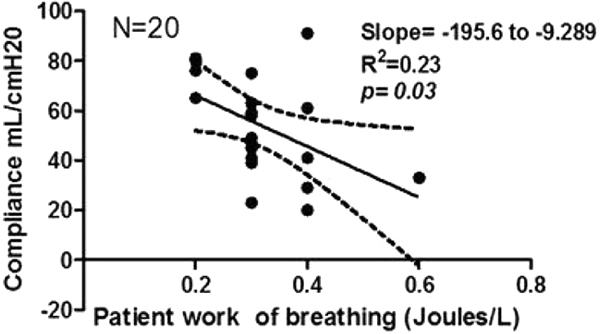
**Correlation between compliance and WOB in PAV-plus**.

Twenty ICU patients recovering from acute respiratory failure were studied, mean age 71.7 ± 9 years, 12 females. Mean minute ventilation at 15 cmH_2_O of pressure support ventilation was 8.4 ± 2.0 l/minute and mean PaCO_2 _was 36.8 ± 5.96 mmHg. Mean minute ventilation was maintained at 8.5 ± 2.0 and 9.48 ± 3.0 l/minute in PAV-plus of 65% and 50%, respectively (*P *= NS). Mean PaCO_2 _was 39 ± 6.6 mmHg in PAV-plus of 65% and 40.65 ± 6.8 mmHg in PAV-plus of 50% (*P *= NS). During PAV-plus 65% the mean estimated patient work of breathing was 0.3 ± 0.1 J/l, and in PAV-plus 50% was 0.4 ± 0.1 J/l (*P *= NS). Mean compliance during PAV-plus 65% was 53.8 ± 20.4 and PAV-plus 50% was 55.3 ± 18.8 (*P *= NS). Mean respiratory resistance was 9.6 ± 4.2 in PAV-plus 65% and 8.7 ± 3.3 in PAV-plus 50% (*P *= NS). Mean comfort scale was 8.45 ± 1.8 in PSV of 15 cmH_2_O and 8.1 ± 1.4 in PAV-plus 65% and 8.1 ± 1.2 in PAV-plus 50% (*P *= NS). Patient's estimated work of breathing significantly associated with respiratory resistance (*P *< 0.0001; Figure 1) and inversely with respiratory compliance (*P *= 0.03; Figure 2) and was not associated with the comfort scale (*P *= 0.8), minute ventilation (*P *= 0.5), PaCO_2 _levels (*P *= 0.5), tidal volume (*P *= 0.3) or respiratory rate (*P *= 0.8).

## Conclusion

ICU patients recovering from acute respiratory failure could be maintained comfortably in PAV-plus ventilation of 65% and 50% compared with PSV of 15 cmH_2_O and their estimated work of breathing correlated negatively with patient's compliance and positively with patient's resistance.

